# A Cross-Sectional Characterization of Insulin Resistance by Phenotype and Insulin Clamp in East Asian Americans with Type 1 and Type 2 Diabetes

**DOI:** 10.1371/journal.pone.0028311

**Published:** 2011-12-02

**Authors:** William C. Hsu, Eyiuche Okeke, Sophia Cheung, Hillary Keenan, Tracy Tsui, Kyle Cheng, George L. King

**Affiliations:** 1 Asian American Diabetes Initiative, Joslin Diabetes Center, Boston, Massachusetts, United States of America; 2 Dianne Nunnally Hoppes Laboratory for Diabetes Complications, Joslin Diabetes Center, Boston, Massachusetts, United States of America; University of Padova, Medical School, Italy

## Abstract

**Objective:**

Classic features of type 1 and type 2 diabetes may not apply in Asian Americans, due to shared absence of common HLA DR-DQ genotype, low prevalence of positive anti-islet antibodies and low BMI in both types of diabetes. Our objective was to characterize diabetic phenotypes in Asian Americans by clamp and clinical features.

**Materials/Methods:**

This was a cross-sectional study conducted in a referral center. Thirty East young Asian American adult volunteers (27.6±5.5 years) with type 1, type 2 diabetes or controls underwent hyperinsulinemic euglycemic clamp to assess insulin resistance and DEXA to assess adiposity.

**Results:**

Gender, BMI, waist/hip ratio, leptin, LDL, anti-GAD, anti-IA2 antibodies and C-reactive protein were similar among three groups. Serum C-peptide, adiponectin, free fatty acid, HDL concentrations and truncal fat by DEXA, were different between diabetic groups. Glucose disposal rate by clamp was lowest in type 2 diabetes, followed by type 1 diabetes and controls (5.43±2.70, 7.62±2.59, 8.61±2.37 mg/min/kg, respectively, p = 0.001). Free fatty acid concentration universally plummeted during steady state of the clamp procedure regardless of diabetes types in all three groups. Adipocyte fatty acid binding protein in the entire cohort (r = −0.625, p = 0.04) and controls (r = −0.869, p = 0.046) correlated best with insulin resistance, independent of BMI.

**Conclusions:**

Type 2 diabetes in Asian Americans was associated with insulin resistance despite having low BMI as type 1 diabetes, suggesting a potential role for targeting insulin resistance apart from weight loss. Adipocyte fatty acid binding protein, strongly associated with insulin resistance, independent of adiposity in the young Asian American population, may potentially serve as a biomarker to identify at-risk individuals. Larger studies are needed to confirm this finding.

## Introduction

The prevalence of diabetes among developed Asian countries is higher than countries in Europe or North America [1]. This is consistent with Asian Americans (AA) experiencing a higher prevalence of diabetes than Caucasians in the United States. In 1983, diabetes prevalence was approximately 20% in second-generation Japanese American men 45–74 years old, compared to 12% Caucasian American men of comparable age [2]. In 2004, 16% of Asian American adults in New York City had diabetes and nearly 45% had either diabetes or pre-diabetes [3], providing more recent evidence that diabetes has become a major public health challenge in the AA community. Since it has been observed that there are multiple clinical and anthropometric features of diabetes that are different between Asians and other ethnic groups, it is not clear whether known clinical characteristics that define type 1 from type 2 diabetes in the Caucasian population would be applicable to Asians or AA. Characterizing the features of different diabetic types in AA sheds important insight into the pathophysiology of diabetes and is crucial for clinicians to provide more tailored and effective care in the diagnosis and treatment of diabetes for this population.

Asians living in the Western Pacific region have the world's lowest prevalence of type 1 diabetes [1]. Uniquely, positivity of auto-antibodies to islet cell antigens is only found in a minority of the newly diagnosed Asians with type 1 diabetes [4], limiting the clinical utility of antibody testing for differentiating diabetic type. Furthermore, specific HLA DR and DQ genotype typically associated with type 1 diabetes is not common in this population [5]. Further diagnostic ambiguity arises from findings that Asians and AA with type 2 diabetes present with a lower and often normal BMI [6] and have younger onset of disease [7], as often found in type 1 diabetes. These unusual characteristics of diabetes in Asians not only render the differentiation of diabetic types particularly difficult in clinical setting especially in younger adults but also suggest that there may be endogenous factors that are different with regard to insulin resistance (IR) in Asians and AA. Final diagnosis often results from clinical observation for ketoacidosis, status of insulin requirement, aided by c-peptide concentration under appropriate clinical situations.

Studies using imaging techniques like DEXA and CT scan have shown that Asian Americans have a higher percentage of visceral fat relative to BMI [8] compared to Caucasians. Even with lower BMIs, IR may be more severe in some of the Asian American populations. Using hyperinsulinemic euglycemic clamp (HEC) in healthy and normal weight individuals matched for BMI, Asian Indian living in the U.S. may be more insulin resistant than Caucasians [9]. However, within the Asian group, it is unclear if IR is different across type 1, type 2 diabetes and controls, given that individuals from all of these groups may all look phenotypically lean. This question has important clinical implications to the Asian and AA population because the typical anthropometric parameters such as BMI and body weight do not accurately reflect degrees of IR and the associated risks for metabolic diseases [10]. If IR is found to be associated with either type of diabetes, it would be important to determine the impact of addressing IR independent of weight loss as advising weight loss may not be appropriate for those who already have normal weight.

Inflammatory and endocrine markers, such as CRP, and other adipose cytokines have been linked to IR although many of these assays generate high degree of variability in the test results and are more suitable for large epidemiologic studies. More recently, retinol binding protein-4 (RBP-4), produced by the adipocytes and liver, has been shown to correlate with insulin resistance in Chinese [11], [12]. In addition, another adipokine, A-FABP has been shown to correlate with HOMA-IR in a Chinese population [13], [14] but has not been correlated with the gold standard measurement of IR like HEC.

Given these unusual pathophysiologic features and diagnostic ambiguity of diabetic types, in this pilot study, we set out to characterize clinical phenotype in AA with type 1, type 2 diabetes and healthy controls and conducted a direct comparative analysis of IR by HEC and contrasted results to conventional and novel biomarkers for IR.

## Materials and Methods

### Ethics Statement

The Institutional Review Board at Joslin Diabetes Center approved this study. Written informed consent was obtained from all participants and all investigation was conducted according to the principles expressed in the Declaration of Helsinki.

The AA subjects in this study included volunteers with type 1 and 2 volunteers, according to the diagnoses given to them by their health care providers, and healthy controls between 18–40 years of age. All participants were of East Asian descendents only (Chinese, Japanese, Korean). Exclusion criteria included history of symptomatic coronary artery disease, peripheral vascular disease, hypertension, hypertriglyceridemia (>500 mg/dl), microalbuminuria, active smoking, daily aspirin, ACE inhibitors, thiazolidinedione, supplementation of vitamin C or E beyond the recommended daily allowance, blood transfusion in the previous three months and pregnant/nursing females. Healthy controls did not have a personal or family history of diabetes in their first-degree relatives. Subjects who fulfilled demographic and diagnostic criteria were identified via our clinic's patient database and recruited from community health centers, college campuses, and fliers.

Subjects underwent phlebotomy, anthropometric measurements, DEXA to determine adiposity and HEC to determine insulin sensitivity. Subjects were instructed to arrive after a 10–12 hour fast. For type 2 diabetes, oral anti-diabetic agents were continued until the day before the study day. For type 1 diabetes, insulin regimen was changed from long acting to short acting formulation the day prior to minimize exogenous insulin before the start of the procedures. Collection for laboratory tests was done on the morning of the study day. Markers of metabolic regulation, insulin release and inflammation analyzed included C-peptide, insulin, free fatty acid, C-reactive protein, adiponectin, RBP-4 (ELISA - ALPCO Diagnostics, USA) and A-FABP (ELISA - Cayman Chemical, France). Anti-islet antibodies measured included glutamate decarboxylase (GAD), islet antigen-2 (IA2) and a competitive insulin antibody assay. DEXA (Hologic 8000) was performed to evaluate total and regional body fat mass.

HEC was used to assess insulin sensitivity. Dextrose solution (20%) was administered via intravenous catheter. A second catheter was inserted retrograde into a vein distal to the first and then placed into a box heated to 70°C for arterialization of venous blood. Following collections of baseline samples, a primed-continuous infusion of regular human insulin (Lilly, Indianapolis) at 80 mU/m^2^/min for 180 minutes was initiated. Blood glucose samples were obtained and glucose infusion rates adjusted every five minutes accordingly to maintain a serum glucose concentration of 90 mg/dl. Glucose disposal rate (GDR) was calculated as the mean glucose infusion rate during the steady state in the last 40 minutes of the clamp procedure.

Statistical Analysis: All variables were visually and statistically inspected for distribution to determine appropriate statistical methods for analysis. Wilcoxon rank sum analysis was used for two-way comparisons involving continuous variables and Fisher's Exact test was used to examine the relationship of categorical variables. Analyses of variance and tests for linear trends were done using generalized linear models, designating groups as categorical or ordinal as appropriate. Linear regression analysis was used to determine correlation coefficients and to adjust for potential confounders. p≤0.05 was considered statistically significant. SAS v. 9.2 (Cary, NC) and STATA 9 (College Station, TX) were used to perform analyses.

## Results

The baseline characteristics of all study subjects (n = 30) are presented in Table 1. Two subjects had history of misdiagnosis. One subject initially presented with hyperglycemia in his early twenties and with a BMI of 24 kg/m^2^. Although he had no history of ketoacidosis, he was given a diagnosis of type 1 diabetes by his original physician given his young age and low BMI. The diagnosis was later corrected to type 2 diabetes by his consulting physician when it was determined that patient had never experienced ketoacidosis even after months of insulin omission. This corrected diagnosis of type 2 diabetes was later confirmed after being on oral agents for many years prior to entry into the study with an elevated c-peptide concentration at the time of the study visit. Another subject was given a diagnosis of type 1 diabetes in her early twenties also because of her young age and a low BMI (<24 kg/m^2^) but had never experienced ketoacidosis nor had a history of positive anti-islet antibodies. She had an elevated concentration of c-peptide 5.72 ng/ml with a fasting glucose of 140 mg/dl 6 years post diagnosis at the time of the study visit. Due to her elevated c-peptide status 6 years after diagnosis, the absence of DKA, the study investigators consulted with her treating physician. A switch of therapy from multiple daily injections to oral agents was made with success, confirming that this subject indeed had been misdiagnosed. The final study sample consisted of East AA adults with type 1 diabetes (n = 10), type 2 diabetes (n = 9), and the non-diabetic control group (n = 11).

**Table 1:** Baseline characteristics, separated by group.

	Type 1 Diabetes (n = 10)	Type 2 Diabetes (n = 9)	Controls (n = 11)	ANOVAp value	Wilcoxonp value
Age (yrs)	25.4±4.5	31.7±6.3	26.3±4.3	0.023	0.0331
Gender male (%)	3(30%)	3(33.3)	5(45.5)	0.74	0.876
Years with DM (yrs)	6.1±4.0	3.0±3.4	N/A	N/A	0.0764
BMI (kg/m^2^)	23.4±1.7	24.5±3.6	23.3±3.9	0.650	0.4965
A1C%	6.9±1.1	7.0±1.6	5.2±0.3	0.001	0.1877
Waist Circumference (cm)	76.7±5.1	84.1±9.8	79.8±10.5	0.200	0.1110
Waist to Hip Ratio	0.85±0.02	0.89±0.53	0.89±0.07	0.150	0.055
Adiponectin (µg/ml)	16.6±5.6	7.3±3.5	8.6±5.1	<0.0001	0.0003
A-FABP (ng/ml)	12.2±3	14.3±3	13.1±6	0.63	0.19
Alb/Creat Ratio (µg/mg)	9.8±15.3	11.3±11.5	9.2±8.6	0.438	0.4140
Alkaline Phosphate (IU/l)	52.2±16.9	41.2±8.6	45.4±9.2	0.157	0.1306
ALT (IU/l)	18.2±4.2	21.2±9.2	20.8±14.9	0.794	0.6521
AST (IU/l)	21.2±6.0	20.3±5.4	20.9±5.0	0.941	0.8694
Diastolic Pressure (mmHg)	76.8±9.1	76.9±7.6	73.9±8.4	0.660	0.68
Systolic Pressure (mmHg)	117.2±17.3	116.2±11.6	109.6±11.6	0.400	0.90
Cholesterol Total (mg/dl)	164.0±43.4	168.1±32.7	167.2±29.4	0.965	0.3911
Cholesterol LDL (mg/dl)	91.6±41.1	103.3±24.2	102.5±24.6	0.648	0.21
Cholesterol HDL (mg/dl)	60.8±10.6	47.8±16.1	47.5±10.7	0.038	0.045
Triglycerides (mg/dl)	65.8±34.7	84.8±44.2	91.6±56.8	0.440	0.2360
C-peptide (ng/ml)	0.14±0.15	2.29±1.57	1.35±1.21	0.001	0.0003
Creatinine (mg/dl)	0.82±0.13	0.73±0.14	0.79±0.14	0.403	0.2415
CRP (µg/ml)	2.68±2.35	1.31±1.11	0.814±1.02	0.048	0.3074
FFA (mEq/l)	0.54±0.19	1.09±0.35	0.77±0.26	0.0003	0.0373
GDR (mg/min/kg)	7.62±2.59	5.43±2.7	8.61±2.37	0.032	0.0942
GFR (mL/min/1.73 m^2^)	101.0±14.7	112.2±21.1	111.0±22.1	0.391	0.1910
HOMA-IR	N/A	2.15±1.95	1.43±0.75	N/A	N/A
Leptin (ng/ml)	10.7±8.4	11.2±7.4	10.2±5.8	0.953	0.9674
RBP-4 (µg/ml)	14.8+4	18.6+6	22.2+8	0.02	0.13
Total Body Fat (%)	23.8±8.4	27.5±5.4	25.9±5.4	0.476	0.6830
Total Body Fat (kg)	14.4±4.7	19.2±6.3	16.8±5.7	0.193	0.0942
Total Body Lean (kg)	45.7±8.7	47.7±9.7	45.7±11.1	0.607	0.4965
Trunk Fat (%)	21.5±8.0	28.8±6.7	25.3±6.2	0.094	0.0942
Trunk Fat (kg)	6.1±2.1	10.3±4.3	8.0±3.7	0.046	0.016
Trunk Lean (kg)	22.4±3.9	23.9±4.8	22.4±5.7	0.691	0.3913
Urea Nitrogen (mg/dl)	14.8±2.5	12.6±3.2	14.1±4.4	0.384	0.1002
GAD Ab+ Subjects*	3(30%)	0%	0%	0.079‡	0.9024
IA2 Ab+ Subjects*	3(20%)	0%	0%	0.23‡	0.88

Data are means ± SD or n (%). ANOVA performed between all 3 groups: Type 1 Diabetes, Type 2 Diabetes, and Controls.

*Auto-antibody positivity to islet cell antigens was determined by serum concentration>0.1 nU/ml for GAD & IA2, expressed as number of individuals (percent positive).

‡ Chi-Square tests were performed in these categories.

Among the three groups, the significant differences included age, adiponectin, C-peptide, FFA, HbA1c, truncal fat by DEXA, GDR, CRP, HDL and RBP-4 (Table 1). The combined diabetic group had a disease duration of 4.6±3.9 years, were in good glycemic control (HbA1c = 6.9±1.3%) and did not have a history of retinopathy, neuropathy or nephropathy. Among those with type 2 diabetes, three were on metformin alone, one was on glyburide alone, one was on a combination of metformin and glyburide, one was on insulin and three were diet controlled.

Subjects with type 1 diabetes compared with subjects with type 2 diabetes had significantly lower C-peptide levels (0.14±0.15 vs. 2.29±1.57 ng/ml, p = 0.0003), higher adiponectin (16.6±5.6 vs. 7.3±3.5 ug/ml, p = 0.0003), higher HDL cholesterol (60.8±10.6 vs. 47.8±16.1 mg/dl, p = 0.045) and only 30% of the subjects with type 1 diabetes had positive anti-GAD antibody titer (30% vs. 0, p = 0.9024). The outstanding features for subjects with type 2 diabetes, compared with the type 1 diabetes cohort were older age (31.7±6.3 vs. 25.4±4.5 yrs, p = 0.0331), higher FFA concentration (1.09±0.35 vs. 0.54±0.19 mEq/l, p = 0.0373), higher truncal fat (10.3±4.3 vs. 6.1±2.1 kg, p = 0.016) and a lower GDR (5.43±2.7 vs. 7.62±2.59 mg/min/kg, p = 0.0942). In contrast, BMI, LDL, waist circumference, CRP, leptin, A-FABP and RBP-4 did not differentiate the types of diabetes (Table 1).

According to HEC, the type 2 diabetic group had the lowest GDR (5.43±2.7 mg/min/kg) followed by the type 1 diabetic group (7.62±2.59 mg/min/kg) and healthy controls (8.61±2.37 min/kg). Both type 1 and 2 diabetic groups had well-controlled and comparable HbA1c (6.9±1.1% vs. 7.0±1.6%, p = 0.19), minimizing the impact of glycemic control on measured insulin sensitivity. The type 2 diabetic group was significantly more insulin resistant compared to the type 1 diabetic and control groups combined (p = 0.01). No significant difference in insulin sensitivity between type 2 diabetic and control groups was found when using the HOMA-IR model (p = 0.32). As expected, 30% of the subjects in the type 1 diabetic group had either positive anti-GAD or anti-IA2 antibodies after an average of 6.1±4.0 years of diabetes.

In contrast to glucose disposal during HEC, FFA disposal as reflected by serum FFA concentration plummeted in all three groups: For controls (0.87±0.29 mEq/L at 0 min, 0.17±0.06 mEq/L at 60 min, 0.12±0.04 mEq/L at 160 min), for type 1 diabetic group (1.36±0.33 mEq/L at 0 min, 0.15±0.05 mEq/L at 60 min, 0.10±0.05 mEq/L at 160 min), for type 2 diabetic group (1.06±0.37 mEq/L at 0 min, 0.20±0.08 mEq/L at 60 min, 0.14±0.13 mEq/L at 160 min).

**Figure 1:**
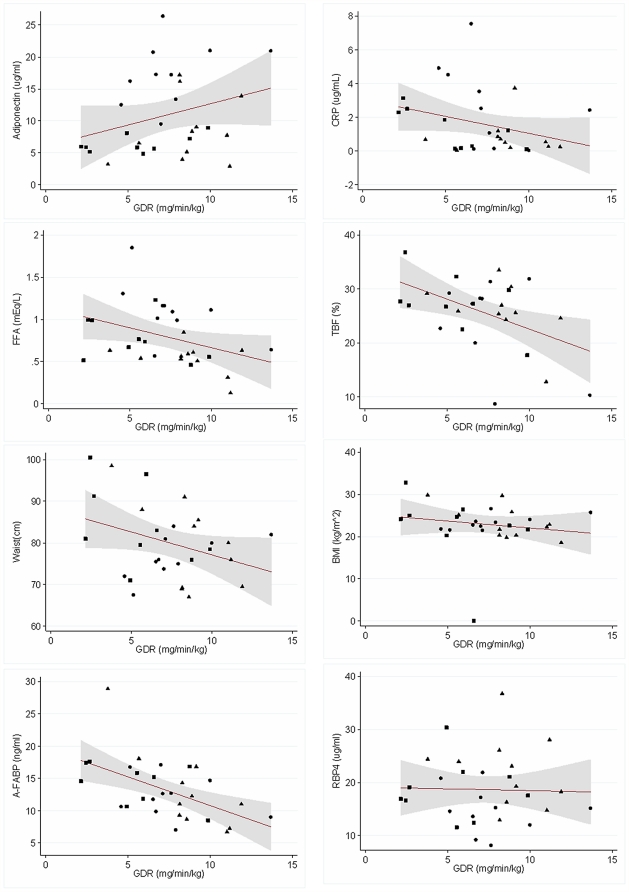
Relationship between glucose infusion rate (GDR) and various biomarkers for insulin resistance. Relationship between GDR and Adiponectin, Free fatty acid (FFA), Waist circumference, A-FABP, C-reactive protein (CRP), Total Body Fat (TBF) by DEXA, BMI, and Retinol Binding Protein-4 (RBP-4). Circles = Type 1 group; Squares = Type 2 diabetes group; Triangles = Control group.

Visual inspections of the scattered plots outlining the relationships between GDR and the biomarkers traditionally associated with IR were performed (Fig. 1). We then conducted linear regression analysis of the relationship between GDR and the parameters of interest. For all three groups combined, A-FABP (r = −0.54, p = 0.002), truncal fat percentage (r = −0.52, p = 0.004), total body fat percentage (r = −0.47, p = 0.01), and BMI (r = −0.36, p = 0.051) correlated with GDR in descending order (Table 2). In contrast, CRP (r = −0.31, p = 0.09) and RBP-4 (r = −0.03, p = 0.87) did not correlate with GDR. For the type 1 diabetic group, the only parameter that correlated with GDR was BMI (r = 0.67, p = 0.03). In the type 2 diabetic group, only CRP correlated with GDR (r = −0.76, p = 0.01). In the control group, A-FABP (r = −0.82, p = 0.02) and BMI (r = −0.62, p = 0.04) correlated with GDR. Because all the variables tested are related to degree of adiposity, we adjusted the analysis for BMI, in addition to age and gender. After adjustment, only A-FABP correlated strongly to GDR in the entire combined group (r = −0.625, p = 0.04) and in controls (r = −0.869, p = 0.046). No other variables showed statistical correlation to GDR in the combined group or in the subgroups.

**Table 2:** Gross and Adjusted Correlations with GDR.

	CRP		A-FABP		RBP-4		TF%		TBF%	
	R_corr_	P_corr_	R_corr_	P_corr_	R_corr_	P_corr_	R_corr_	P_corr_	R_corr_	P_corr_
All	−0.36	0.051	−0.31	0.09	−0.54	0.002	−0.03	0.87	−0.52	0.004
Type 1	0.67	0.03	−0.4	0.24	−0.29	0.42	−0.2	0.58	−0.4	0.25
Type 2	−0.54	0.13	−0.76	0.01	−0.49	0.18	0.01	0.98	−0.52	0.15
Controls	−0.62	0.04	0.01	0.98	−0.82	0.002	−0.21	0.5	−0.55	0.1
Adjusted Correlations with GDR by age, gender and BMI										
All	−0.588	0.107	−0.625	0.04	−0.528	0.637	−0.553	0.144	−0.452	0.112
Type 1	−0.762	0.794	−0.763	0.774	−0.822	0.266	−0.759	0.877	−0.76	0.85
Type 2	−0.797	0.391	−0.745	0.84	−0.746	0.805	−0.742	0.953	−0.756	0.682
Controls	−0.707	0.901	−0.869	0.046	−0.722	0.612	−0.779	0.295	−0.835	0.13

Data are presented as Pearson correlation coefficient (R), and corresponding p value. Variables presented: BMI, C-Reactive Protein (CRP), Adipocyte Fatty Acid-Binding Protein (A-FABP), Retinol Binding Protein-4 (RBP-4), Total Body Fat (TBF%) by DEXA, and Truncal Fat percentage (TF%). Type 1 diabetes group (Type 1), Type 2 diabetes group (Type 2). Adjusted correlations with GDR by age, gender and BMI.

## Discussion

This study provided the first comparative analysis of clinical phenotype of type 1 and 2 diabetic subjects and healthy controls in AA. This study confirms that type 1 and type 2 diabetes in AA share many similar physical characteristics but retain differences fundamental to their respective pathophysiology. In spite of having comparable BMIs and waist circumferences, our results from HEC suggested that type 2 diabetes in AA is more insulin resistant compared with type 1 diabetes and healthy controls. A-FABP was identified as having the strongest correlation to IR compared to conventional markers in AA.

Although there is no reliable data on the frequency of clinical misdiagnosis of diabetes type in AA, two individuals with type 1 diabetes in our study were initially misdiagnosed by their physicians, illustrating the diagnostic challenge in young AA. Markers of autoimmunity to islet cells may not be helpful as studies in Asians found that less than half have evidence of auto-antibodies against islet antigens at diagnosis [4], which is usually positive in 90% in Caucasian patients at time of diagnosis [15]. Commonly used clinical measurements such as BMI and other inflammatory biomarkers did not differentiate types of diabetes in AA which are different from studies involving Caucasian and other minority populations that show BMI and CRP are reliable predictors of type 2 diabetes [16]. Clinical diagnosis based on status of insulin dependence, history of ketoacidosis aided by unequivocal c-peptide concentration often guides clinical decision in many ambiguous cases. In addition, parameters such as adiponectin, HDL, FFA concentrations, truncal fat and GDR, not only differentiated type 2 from type 1 diabetes, but also suggested a mechanistic link to visceral adiposity. The elevated levels of adiponectin in AA with type 1 diabetes are interesting and clearly differentiated them from AA with type 2 diabetes. Further studies in Asian or AA populations are needed to confirm whether this significant elevation of adiponectin in AA with type 1 diabetes is related to young age, good glycemic control and the low prevalence of cardiovascular complications in East AA.

An earlier study found that healthy non-obese AA matched for body fat percentage are more insulin resistant than their Caucasian counterparts using the hyperglycemic clamp method [17]. Our study using HEC extended the finding within the East AA ethnic group that IR is a pathophysiological component of type 2 diabetes even when their weight is within normal range. Future studies are needed to determine whether targeting insulin resistance independent of weight loss is important in the treatment of type 2 diabetes in this population. Furthermore, this study provides new data to document, using HEC and supported by high HDL and adiponectin, that AA with type 1 diabetes do not have significant IR if glucose levels are well controlled. The positive correlation between GDR and BMI in the type 1 diabetic group (Figure 1) is worth noting. Although the mechanism is unclear, the correlation could be related to the degree of glycemic control in type 1 diabetes as optimal glucose control improves insulin sensitivity but may lead to weight gain.

Unlike the variable glucose disposal rates among the three groups, a sharp decline in FAA concentration was universal during HEC in all three groups. Insulin is known to promote the synthesis of fatty acids in the liver and reduce the breakdown of fat in the adipose tissue by inhibiting hormone sensitive lipase activities. Despite the known fact that FAA are elevated in type 2 diabetes, the high insulin concentration used during HEC obliterated the differences in FAA but not glucose disposal among the three groups, raising the likely possibility that that regional insulin resistance towards glucose metabolism not fat metabolism, may underlie the pathophysiology of IR in AA.

Using HEC, we have documented that A-FABP is closely associated with IR but mostly in the control group and not so tightly once diabetes has occurred. This supports that IR has a greater effect on FABP levels in non-diabetic controls than in the diabetic state and suggests that other factors besides IR regulates FABP once diabetes has developed. The strong correlation of A-FABP to IR, independent of BMI, may have a clinical relevance to screening for risks of diabetes in AA because generalized obesity is not common and a blood test is simple for assessing IR. Furthermore, the existing correlation independent of BMI also suggests that A-FABP levels may not be regulated by pathways related to obesity. A-FABP, a cytoplasmic protein abundantly present in serum and expressed only in adipocytes and macrophages, avidly binds to intracellular fatty acids [18]. Their functions include the transport of FFA to cytoplasmic compartments in addition to regulating gene expression relating to lipid metabolism and inflammatory cytokines. FABP has been shown to be regulated by glucocorticoids, PPAR- γ agonists, fatty acids and insulin [19], which may provide the mechanic framework for the link to insulin sensitivity. In a longitudinal studies from Hong Kong, serum A-FABP was associated with glucose intolerance and predicted the development of type 2 diabetes in a Chinese cohort followed over ten years [14], pointing to its potential as a prognostic tool. Similarly, A-FABP was associated with metabolic syndrome independent of adiposity and IR, expanding its role as a predictor for cardiovascular diseases [13]. However, our results, although limited by the small sample size, raises the hypothesis that diagnostics targeting A-FABP may only be effective before the onset of diabetes.

In contrast to earlier studies, we did not find RBP-4 to be correlated to GDR. The original publication linking RBP-4 to IR was done in Caucasians only [11]. Our study also differed from the conclusion of a study from China [12] showing a correlation of RBP-4 to IR, in that we studied AA and used a different assay. The major weakness of the study is the small sample size which limited our ability to run multiple adjustments in the model regarding smoking, family history and physical activities. It also did not allow a comprehensive matching between the groups. Stopping oral anti-diabetic agents prior to the entry of the study would have been an ideal way to study IR in the type 2 group. However, the internal review board discourages such practice due to putting patients at risk for adverse events even if the risk is minimal. To address the potential effect of oral agents, we have specifically excluded individuals on thiazolidinediones, which are known to have the most impact on insulin resistance, to minimize the impact of oral agents on measured insulin sensitivity. In addition to determine IR, assessing the β-cell function in non-obese individuals with type 2 diabetes is very important, however, beyond the scope of this pilot study. Therefore a separate study will be needed to fully examine the interaction between insulin secretion and insulin resistance. It is likely that our result only applies to young East AA populations. South Asian Americans are generally more insulin resistant [20], have elevated CRP concentrations and more cardiovascular diseases despite sharing the similar feature of low BMI with East AA. The strength of our study is the use of HEC as the definitive procedure for the assessment of insulin sensitivity, enabling the direct comparison of emerging biomarkers with conventional markers for IR. We have also included the assessment of IR in type 1 diabetes in East AA subjects, which has not been reported in previous literature.

In summary, our pilot study confirmed that insulin resistance is a pathophysiologic feature of type 2 diabetes in AA despite the association with normal BMI. Biomarkers, such as adiponectin, that reflect visceral adiposity maybe more sensitive than conventional anthropometric markers in differentiating type 1 from type 2 diabetes in AA. Lastly, A-FABP concentration, seemingly unaffected by adiposity, emerged as an interesting biomarker for IR and may be useful for identifying non-diabetic AA with IR.
